# Can the posterior:anterior urethral ratio on voiding cystourethrogram be used as a reliable predictor of successful posterior urethral valve ablation in male children?

**DOI:** 10.4102/sajr.v24i1.1820

**Published:** 2020-06-09

**Authors:** Zakiyah Gaibie, Nasreen Mahomed, Karen L. Petersen, Glenda Moonsamy, Akram A.H. Bokhari, Ahmed Adam

**Affiliations:** 1 Division of Urology, Department of Surgery, School of Clinical Medicine, Faculty of Health Sciences, University of the Witwatersrand, Johannesburg, South Africa; 2 Department of Urology, Helen Joseph Hospital, Johannesburg, South Africa; 3 Department of Paediatric Urology, Rahima Moose Mother and Child Hospital, Johannesburg, South Africa; 4 Department of Radiology, School of Clinical Medicine, Faculty of Health Sciences, University of the Witwatersrand, Johannesburg, South Africa; 5 Division of Paediatric Nephrology, Department of Paediatrics and Child Health, School of Clinical Medicine, Faculty of Health Sciences, University of the Witwatersrand, Johannesburg, South Africa; 6 Department of Paediatric Nephrology, Chris Hani Baragwanath Hospital, Johannesburg, South Africa; 7 Department of Paediatric Nephrology, Charlotte Maxeke Academic Hospital, Johannesburg, South Africa; 8 Department of Urology, College of Medicine, Hail University, Hail, Saudi Arabia; 9 Department of Urology, King Fahad General Hospital, Jeddah, Saudi Arabia

**Keywords:** Posterior urethral valves, Urethral ratio, Posterior anterior urethral ratio, Voiding cystourethrogram, Golden ratio

## Abstract

**Background:**

The role of the voiding cystourethrogram (VCUG) in the follow-up of children with posterior urethral valves (PUVs) post-ablation has been considered a standard practice. The urethral ratio and gradient of change have proven to be useful.

**Objectives:**

We aimed to review the role of the ‘ideal’ ratio on predicting residual PUV post-ablation.

**Methods:**

A systematic review of the PubMed, SCOPUS and Web of Science databases was performed (April 2019). The search terms included ‘Urethral Ratio and Posterior urethral valve ablation’. All cited reference lists were further evaluated for additional inclusive studies.

**Results:**

Eleven studies were identified, of which nine were relevant to the topic. Case reports, comments and adult and animal studies were excluded, leaving four studies for critical review. In total, 338 patients were assessed. The control group consisted of 167 age-matched, male children. Study regions included India and Australia. The ages ranged from 15 days to 3.4 years. Ablation methods included the use of a resectoscope with cutting diathermy, cold knife or Bugbee electrode. The mean urethral ratios in the control group ranged from 1.04 to 1.73. The suggested predictive urethral cut-off ratios recommended include 2.2 (*p* = 0.001), 2.5–3 and 3.5.

**Conclusion:**

Although the precise cut-off ratio could not be clearly defined in this review, a urethral ratio less than a range of 2.2–3.5 has proven to be a beneficial predictor of ablation success and should thus be incorporated into standard VCUG reporting templates in the follow-up of PUVs in male children in resource-limited settings.

## Introduction

**FIGURE 1:**
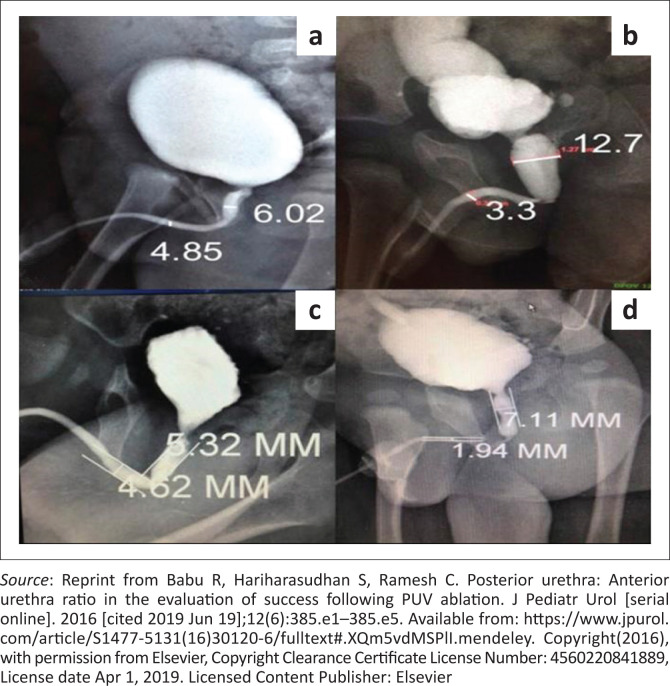
On voiding cystourethrogram (VCU) the posterior urethra:anterior urethra ratio (PAR) was computed by dividing the maximum posterior urethral diameter by the anterior urethral diameter (in mm). Distances were measured by an on-screen distance measurement tool in the radiology department, to avoid error. (a) Normal voiding cystourethrogram – control: PAR = 6.02/4.85 = 1.24; (b) posterior urethral valve (PUV) at the time of diagnosis: PAR = 12.7/3.3 = 3.84; (c) successful PUV ablation: PAR = 5.32/4.62 = 1.15; (d) persistent obstruction because of a stricture or residual valve: PAR = 7.11/1.94 = 3.66.

The role of the voiding cystourethrogram (VCUG) in the follow-up of male children with posterior urethral valve (PUV) ablation has been considered as standard practice in the treatment and follow-up algorithm. Since 2006, the use of the posterior:anterior urethral ratio (PUR) and gradient of change has proven to be beneficial.^1,2,3,4^ The urethral ratio is calculated on standard VCUG images, as shown in Figures 1a–d. In this systematic review, we aimed to determine the ‘ideal’ ratio and assess the role of this ratio in predicting residual PUV in the follow-up of post-ablation male children.

## Methods

To illustrate and better define the role of calculating the urethral ratio as part of routine assessment during the reporting of the VCUG study, a systematic review of the PubMed, Web of Science and SCOPUS databases was performed in April 2019.

The search terms ‘Urethral Ratio and Posterior urethral valve ablation’ were utilised. The cited reference lists of studies were identified and deemed relevant and were further evaluated for additional inclusive studies assessing this parameter.

### Ethical considerations

This article followed all ethical standards for a research without direct contact with human or animal subjects.

## Results

**FIGURE 2:**
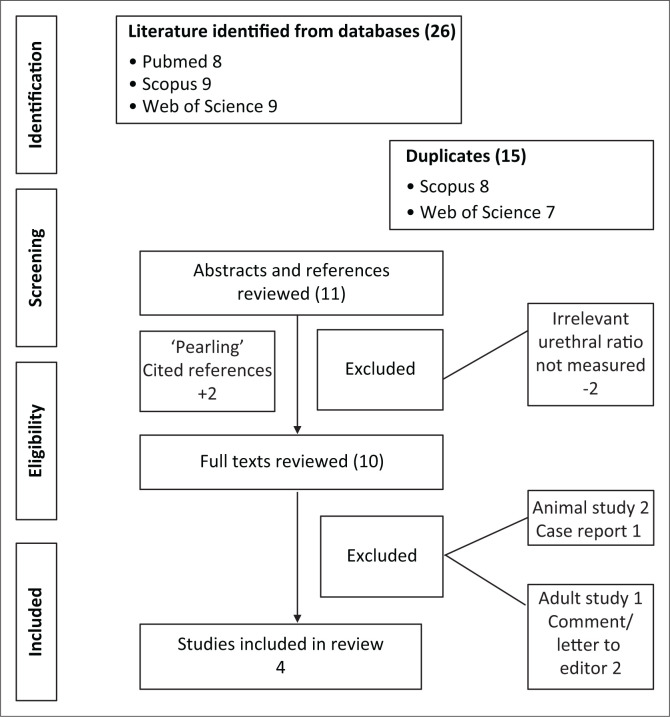
Flow diagram demonstrating how the systematic review of the posterior:anterior urethral ratio on voiding cystourethrogram from the PubMed, Web of Science and SCOPUS databases was performed.

Eleven studies were identified using search terms ‘Urethral Ratio and Posterior urethral valve ablation’. There were 10 full-text reviews, of which two were animal studies, one was a case report, one was an adult study and two were letters to the editor; these were excluded (Figure 2). The remaining four prospective studies in male children were evaluated in detail and the results are tabulated in Table 1.

**TABLE 1:** Tabulation of studies assessed within this review.

Author	Region (year)	Study period	Number of patients	Age (median)	Single surgeon [±]	Control group	PAR ratio control group (SD)	Pre-ablation ratio (SD)	Post- ablation group (SD)	Number requiring second ablation	Ratio prior to second ablation Mean	Method of ablation performed (cold knife/ablation/laser/not mentioned)	Follow-up VCUG	Study cut-off ratio	Author’s conclusion
**Bani Hani et al.**	Westmead, Australia (2006)	1995–2004	35 (PUV patients)	23 <1 year Median 1.5 months	Single	31 (VCUG for UTI)	Median 2.6	*N* = 13 Median 8.6	*N* = 15 Median 3.1	5	8	11 Fr resectoscope with Collins knife/cutting Diathermy or 9 Fr cystoscope with 2.4 Fr electrode	6–8 weeks	3.5	Ratio is an objective tool in assessing success of valve ablation
**Gupta et al.**	Mumbai, India (2009)	2005–2007	30	Median 13 months	Not mentioned	30	Mean 1.73 (0.577)	Mean 4.94 (2.97)	Mean 2.134 (1.19)	2	5.5 (5 and 6)	10 Fr resectoscope or 7.5 Fr cystoscope with Bugbee electrode	3 months	2.5–3 (*p* < 0.001)	PUR is an objective and reproducible method of assessing PUV patients post ablation
**Menon et al.**	Chandigarh, India (2010)	2004–2010	217 A 133 B 11 C 16 D 21 E 36	Mean A 10 months B 3.4 years (7 months) C 10 months D 27 months	Single	50	Mean 1.04 (0.293)	Mean A 5.82 B 12.269 C 10.5	Mean A 1.026 (<2 SD) B 1.734 (2–3 SD) C 3.69 (>SD) D 1.82	A 0 B 0 C 8 D 21	C 4.51 D 7.92	9 Fr resectoscope, hook electrode and cutting diathermy	3 months	3	Good clinical outcomes in patients who showed normalisation of PU. Strong correlation of persistent symptoms with PUR that remains high
**Babu et al.**	Chennai, India (2016)	2013–2016	56	3–250 days/median 15 days	Single	56, age-matched	Mean 1.5 (0.42)	Mean 3.42 (0.75)	Mean 1.8 (0.21)	5	3.16 (0.54)	8.5/9 Fr cystoscopy and cold knife	Routine 3 months	2.2 (*p* = 0.001)	Ratios >2.2 require cystoscope (residual valve vs. stricture)

SD, standard deviation; VCUG, voiding cystourethrogram; UTI, urinary tract infection; PUV, posterior urethral valve; PUR, posterior anterior urethral ratio; PU, posterior urethra.

Study regions included Mumbai, Chandigarh and Chennai, India, as well as Westmead, Australia.^1,2,3,4^ In total, 338 patients with PUVs were assessed. The control group consisted 167 patients, who were age-matched in most studies.^1,2,3,4^ The urethral ratios, for the control group, were calculated from VCUGs being done as part of the workup for urinary tract infections (UTIs). The mean urethral ratios in these children ranged from 1.04 to 1.73.^2,3,4^ The median urethral ratio for the control group by Bani Hani^1^ was 2.6.

The median age for male children with PUVs ranged from 15 days to 13 months.^1,2,4^ Menon et al.^3^ grouped patients into three groups on the basis of post-operative urethral ratios. Group A consisted of patients whose post-operative urethral ratios were <2 SD from the control group mean, group B between 2 and 3 SD and group C >3 SD from the control mean urethral ratio. The mean age ranged from 10 months to 3.4 years.

A single surgeon was used in three of the four studies to limit confounding factors.^1,3,4^ Based on the surgeons’ preference and patient factors, the methods for ablation included the use of a resectoscope with cutting diathermy, cold knife or Bugbee electrode.^1,2,3^ Mean pre-operative ablation ratios ranged from 3.342 to 12.269. Post-ablation ratios ranged from a mean of 1.026–3.69.^2,3,4^ Other authors observed a median ratio of 8.6 preoperatively and 3.1 postoperatively.^1^ A total of 41 (12%) patients required a second ablation because of ongoing symptoms and residual valves.^1,2,3,4^ The urethral ratio prior to the second ablation ranged from a mean of 3.16 to 8.^1,2,3,4^ Menon et al. demonstrated that in 50% of patients in whom the postoperative urethral ratio was >3 SD of the control group, repeat ablation was required. Only 31.3% of patients in this group were asymptomatic. The cut-off ratios recommended include 2.2 (*p* = 0.001), 2.5–3, 3 and 3.5.^1,2,3,4^

### Method of ratio calculation

All studies emphasise voiding films in oblique views. However, Bani Hani^1^ and Gupta et al.^2^ measured the transverse diameter of the posterior urethra midpoint between bladder neck and distal membranous urethra which may not always be the point of maximal distension, whereas Menon^3^ and Babu^4^ used the maximum diameter of the posterior urethra. In addition, Babu^4^ used an on-screen distance measuring tool. The anterior urethra transverse diameter was measured by all at the maximum distension of the bulbar urethra.^1,2,3,4^

### Meta-analysis

A meta-analysis of the relevant data was precluded in this review study because the studies reviewed were performed by different surgeons, using different instruments, and were reported at different follow-up durations.

## Discussion

The prevalence of congenital PUVs is 1 in 8000 in male children.^5^ Posterior urethral valves account for one of the main causes of renal dysfunction in male children.^6^ Posterior urethral valves cause bladder outlet obstruction because of an abnormality of the membranous urethra.^7^ Diagnosis can be made on antenatal ultrasound. Features include megacystis with a thickened bladder wall and poor emptying on 30-min ultrasound. The presence of oligohydramnios, echogenic kidneys, hydronephrosis and foetal ascites is indicative of a poor prognosis. Diagnosis made before the end of the second trimester is associated with a higher perinatal mortality and end-stage renal disease.^8^

In South Africa, post-natal diagnosis is made within the first year of life in most cases.^9^ This is in contrast to developed countries where the diagnosis is often made at antenatal visits. Male children not only present with UTIs, palpable bladder and palpable kidney,^9^ but may also present with failure to thrive, dehydration, vomiting, fever, electrolyte imbalances and uraemia.^8^ Male children over 5 years old may present with diurnal enuresis and obstructive urinary symptoms.^8^

The current suggested workup includes the following:

- Renal function test and blood gas analysis
- Urinalysis
- Kidney-bladder ultrasound
- Voiding cystourethrogram
- Renal dynamic scan in male children older than a month (catheter to be left in situ if there is hydronephrosis)
- Dimercaptosuccinic acid (DMSA) radionuclide scan to assess scarring in the presence of reflux. It is important that this scan is done 6 months after the last UTI.

Primary management involves first stabilising the child, managing sepsis, obtaining adequate hydration, correcting electrolytes and relieving obstruction.^8^ Once the child is optimised, definitive management is primary ablation of the urethral valves.^8^ Urinary diversion has become less favourable with the availability of adequately sized resectoscopes, and the ability to primarily ablate valves in younger male children.^9^

Children require close follow-up post-ablation. The aims of follow-up are to identify and manage voiding dysfunction early, monitor and optimise renal function, attain continence and minimise infections.^8^ Recommended follow-up intervals are at 3, 6, 9 and 12 months post-ablation and then annually up to the age of 15, depending on the glomerular filtration rate (GFR). Male children with chronic kidney disease (CKD) stages 4–5 are followed up more frequently. Routine follow-up consists of urinalysis, urea, creatinine, electrolytes, uroflowmetry and ultrasound with measurement of any post-void residual urine volume.^8^ A routine VCUG or cystoscope is done at 3 months post-ablation to assess structural anomalies of the bladder, posterior urethra and obstructive lesions such as residual valves and strictures.^8^ There is ongoing debate about the preferred method of assessment of valve ablation adequacy, regarding whether clinical, radiological or cystoscopic assessment is ideal. Proponents for VCUG argue against the need for invasive cystoscopy and general anaesthesia, while proponents for cystoscopy argue against the predictive value of VCUG.^10,11^

International guidelines should be modified or adjusted depending on the resources of the country. Routine post-operative cystoscopy on all patients is not feasible in a developing country. Selecting patients on the basis of clinical and radiological parameters for referral for repeat cystoscopy may be necessary.

In 10% – 30% of male children, there may be residual valves that require a second ablation. Twelve per cent of patients in this review required repeat ablation. Persistent symptoms of poor stream, nocturnal enuresis and persistent vesicoureteric reflux (VUR) may be indicators of residual valves.^12^ Younger age at ablation, echogenic kidneys, hydronephrosis and high-grade reflux preoperatively have been associated with residual valves.^12^ The urethral ratio has proven to be an objective and reproducible method in assessing the success of valve ablation.^1,2^ Male children who showed normalisation of the urethral ratio had good clinical outcomes.^3^ There is a strong correlation with persistent symptoms and a urethral ratio >3 SD from the normal.^3^

Indications for VCUG include recurrent UTI post-ablation, worsening creatinine, assessment for the presence and grade of VUR, persistent or worsening hydro-ureteronephrosis (repeat done at 2, 5 and 10 years) or if further surgical management is being planned, that is, transplant, anti-reflux surgery or bladder augmentation.^8^ Vesico-ureteric reflux is present in up to 50% of male children preoperatively, of which 60% persist postoperatively. High-grade reflux may require anti-reflux surgery, whereas low-grade reflux can be managed medically.^8^

Bladder dysfunction persists in one-third of male patients and causes persistent hydronephrosis and VUR.^8^ In one study, the shape, wall, reflux and diverticuli (SWRD) score was found to be an objective tool in quantifying the severity of bladder dysfunction.^13^ The score was calculated on the basis of shape and wall of the bladder as well as the presence of reflux and diverticuli on cystograms taken during video-urodynamic studies. This had treatment and prognostic implications. Scores >2 were found to be ‘hostile’ bladders requiring invasive intervention, whereas a score <2 was indicative of ‘compliant’ bladders or bladders with low-pressure detrusor overactivity that could be managed medically.^13^ Bladder neck incision (BNI) at the time of valve ablation has been proposed to improve outcomes of obstructive voiding symptoms post-ablation. This practice was previously abandoned as it was thought to cause incontinence, dry or retrograde ejaculation. However, a study by Keihani et al.^14^ did not show ejaculatory dysfunction or incontinence at the follow-up assessments.

Ratios in anatomy have been explored in various previous reports. The concept of the ‘golden ratio’ is well known and has been derived from the Fibonacci sequence, the numerical value being 1.618.^15^ Over the years it has been studied and proven to represent the ‘settings’ of botanic structures, humans and their organ systems.^15^ We have reviewed a ratio in the context of the follow-up VCUG. Despite surgical intervention, renal outcomes may still be poor.^9^ In a retrospective study done by Petersen et al.,^9^ 34.8% of male children developed CKD despite early ablation. In a 5-year follow-up study done by Uthup et al.,^16^ 50% of patients were still symptomatic and 33% of patients had developed CKD and growth failure. Mean time to development of end-stage renal disease was found to be 10.7 years post-ablation.^6^

## Conclusion

The urethral ratio has proven beneficial in the international literature and should be incorporated into standard VCUG reporting templates in the follow-up investigations of PUV in male children. Radiologists reporting on post-PUV ablation VCUG studies are requested to measure and report these ratios. A urethral ratio less than a range of 2.2–3.5 has been shown to be a beneficial predictor of ablation success. Further prospective studies are required to better define an exact cut-off point, especially in resource limited settings.
